# PdAgPt Corner-Satellite Nanocrystals in Well-Controlled Morphologies and the Structure-Related Electrocatalytic Properties

**DOI:** 10.3390/nano11020340

**Published:** 2021-01-29

**Authors:** Hehe Qian, Jianzhou Wu, Yongsheng Guo, Wenjun Fang

**Affiliations:** Department of Chemistry, Zhejiang University, Hangzhou 310058, China; saki@zju.edu.cn (H.Q.); wjzclig@zju.edu.cn (J.W.); wjjw@zju.edu.cn (Y.G.)

**Keywords:** heterogeneous metallic nanocrystals, formic acid oxidation, seed-mediated growth, morphology control

## Abstract

The functions of heterogeneous metallic nanocrystals (HMNCs) can be undoubtedly tuned by controlling their morphologies and compositions. As a less-studied kind of HMNCs, corner-satellite multi-metallic nanocrystals (CSMNCs) have great research value in structure-related electrocatalytic performance. In this work, PdAgPt corner-satellite nanocrystals with well-controlled morphologies and compositions have been developed by temperature regulation of a seed-mediated growth process. Through the seed-mediated growth, the morphology of PdAgPt products evolves from Pd@Ag cubes to PdAgPt corner-satellite cubes, and eventually to truncated hollow octahedra, as a result of the expansion of {111} facets in AgPt satellites. The growth of AgPt satellites exclusively on the corners of central cubes is realized with the joint help of Ag shell and moderate bromide, and hollow structures form only at higher reaction temperatures on account of galvanic displacement promoted by the Pd core. In view of the different performances of Pd and Pt toward formic acid oxidation (FAO), this structure-sensitive reaction is chosen to measure electrocatalytic properties of PdAgPt HMNCs. It is proven that PdAgPt CSMNCs display greatly improved activity toward FAO in direct oxidation pathway. In addition, with the help of AgPt heterogeneous shells, all PdAgPt HMNCs exhibit better durability than Pd cubes and commercial Pt.

## 1. Introduction

Heterogeneous metallic nanocrystals (HMNCs) with controllable sizes, morphologies and compositions are attracting growing interest due to their sophisticated architectures and great application potentials [1]. It has been proven that the functions of HMNCs, such as electrocatalytic performances, are highly dependent on their structures [2,3,4,5,6,7]. Precisely controlling the compositions, structures and morphologies of HMNCs can directly impact their functionalities [8,9]. A body of research demonstrates that electrocatalytic reactions, such as formic acid oxidation (FAO), have reflected the structural characteristics of nanomaterials, especially nanostructures based on Pt and Pd [10,11,12,13,14,15,16,17,18,19,20]. Both of the two noble metals are universal components of electrocatalysts toward FAO. There is a dual-pathway mechanism involved in FAO on Pt [21,22,23,24,25]: (a) the direct pathway through the dehydrogenation reaction without forming the intermediate product, i.e., CO; and (b) the indirect oxidation pathway in which CO forms and requires OH groups to further oxidize CO to CO_2_ at a higher potential. While the FAO on Pd generally follows the direct pathway with low onset potential and high catalytic activity, the poor electrochemical stability of Pd in the acidic environment would greatly undermine electrocatalytic properties [26,27,28].

Designing well-controlled nanostructures with the two noble metals provides an effective strategy to combine their superiorities and avoid defects [29,30,31]. In recent decades, a rich variety of multi-metallic nanocrystals have been fabricated based on Pd and Pt with well-controlled morphologies and compositions [32,33,34,35,36,37,38,39,40,41]. For instance, Tang and his team reported a facile self-assembly strategy to prepare PtPd@Pt core/satellite nanoassemblies as cathodic electrocatalysts [42]. In another report, researchers have realized the selective deposition of satellite AgPt nanocrystals on corners of the central Au octahedra via galvanic displacement [43]. Among all the core/satellite structures, corner-satellite multi-metallic nanocrystals (CSMNCs) are a unique class of HMNCs with high geometrical symmetries and fine nanoarchitectures. Compared with other multi-metallic nanostructures, e.g., core-shell nanostructure, the CSMNCs with higher possibility of new properties or multi-functionality are worth being paid more attention. However, there has been a very limited amount of research into CSMNCs [44]. Generally, the satellite nanocrystals on the specific sites of polyhedral seeds can form and grow by means of site-selective deposition. During this process, morphology control involving both the satellite nanocrystals and central part poses a dual challenge [45,46].

Herein, we propose an easy and convenient approach to morphology-controlled synthesis of PdAgPt corner-satellite nanocrystals via temperature regulation in seed-mediated growth. From well-defined Pd@Ag nanocubes, we obtain four types of PdAgPt HMNCs, especially corner-satellite nanocrystals with a hollow structure. As the reaction temperature rises, the evolution of AgPt octahedral satellites is presented with increasing selective deposition of Pt, and a hollow structure forms due to promoted galvanic displacement. The roles of Ag atoms and bromide anions in the production of PdAgPt HMNCs have been revealed through relatively exhaustive investigations. Thanks to the unique corner-satellite nanostructures, as-prepared PdAgPt CSMNCs show enhanced electrocatalytic activity toward FAO in direct oxidation pathway, compared with Pd cubes and commercial Pt. The combination of Ag-modulated surfaces and hollow structures leads to a higher proportion of the direct oxidation pathway and more negative onset potential. Therefore, this work provides a practical strategy to construct HMNCs to reveal more about structure-related functions and develop high-performance electrocatalysts.

## 2. Materials and Methods

### 2.1. Materials

Potassium tetrachloropalladate (K_2_PdCl_4_, 99.95% metals basis), ascorbic acid (C_6_H_8_O_6_, AA, 99.99% metals basis), polyvinylpyrrolidone ((C_6_H_9_NO)_n_, PVP, MW = 58,000), potassium chloride (KCl, GR, 99.8%), potassium bromide (KBr, 99.95% metals basis), potassium chloroplatinate (K_2_PtCl_6_, 99.9% metals basis), silver trifluoroacetate (CF_3_COOAg, 98%), perchloric acid (HClO_4_, GR, 70.0–72.0%) and potassium iodide (KI, ultra-pure, ≥99.5%) were purchased from Aladdin Industrial Co., Shanghai, China. Nafion solution 117 was purchased from Sigma-Aldrich Co., LLC., Shanghai, China. Carbon black (Vulcan XC-72, Cabot), platinum (II) acetylacetonate (Pt(acac)_2_, 97%) and ethylene glycol (EG, anhydrous grade, 99.8%) were provided by Macklin Biochemical Co., Ltd., Shanghai, China. Isopropyl alcohol, acetic acid, ethanol as well as acetone were obtained from Sinopharm Chemical Reagent Co., Ltd., Shanghai, China. All of these reagents were employed without further purification. Ultrapure water with resistivity of 1.82 × 10^5^ Ω·m at 25 °C was produced from a Millipore Q3 system (Merck, Shanghai, China).

### 2.2. Synthesis of Pd@Ag Core-Shell Nanocubes

Size-controlled Pd nanocubes were basically prepared following the typical synthesis procedure reported before [47]. Pd nanocubes served as seeds were washed with water and acetone five times to eliminate adsorbed substances on the surface, and then re-dispersed with EG. EG solution 2.5 mL containing PVP (32 mg mL^−1^), KBr (13.4 mmol L^−1^) and Pd nanocubes (Pd element, about 10 mmol L^−1^) were preheated in a glass vial under magnetic stirring at 145 °C for 5 min. Then, 100 μL EG solution of CF_3_COOAg (50 mmol L^−1^) was titrated into the vial in half a minute, and the reaction lasted 30 min at 145 °C. Afterwards, the products were collected for Pt selective deposition.

### 2.3. Synthesis of PdAgPt Corner-Satellite Nanocrystals

For the synthesis of PdAgPt nanocrystals in different morphologies, Pt(acac)_2_ was introduced into above-mentioned products (Pt element, 4.8 mmol L^−1^) and the reactions continued for 3 more hours at set temperatures (125 °C, 145 °C, 165 °C, 185 °C). Afterwards, the products were washed with water and acetone three times, and then re-dispersed in 1.5 mL ultrapure water with 10 min of ultrasonic treatment, stored for further characterization.

### 2.4. Characterization

Transmission electron microscopy (TEM) images were captured by HT7700 (Hitachi, Tokyo, Japan) with acceleration voltage at 100 kV, and high-resolution TEM (HRTEM) analyses with energy disperse X-ray (EDX) were obtained on a 200 kV Tecnai G^2^ F20 S-TWIN (FEI, Hillsboro, OR, USA). The atomic compositions were further determined using inductively coupled plasma-optical emission spectrometers (ICP-OES, 730-ES, Varian, Palo Alto, CA, USA). X-ray diffraction (XRD) patterns of the electrocatalysts were recorded with a D8 Advance diffractometer (Bruker, Karlsruhe, Germany). X-ray photoelectron spectra (XPS) were obtained on a ESCALAB 250Xi spectrometer (Thermo, London, UK) with a monochromatic Al Kα X-ray source.

### 2.5. Electrochemical Measurements

All electrochemical measurements were carried out at room temperature in a three-compartment electrochemical cell, conducted by CHI 604D potentiostat (CH, Shanghai, China). Saturated calomel electrode (SCE), platinum plate electrode and pre-treated glassy carbon electrode (GCE, 0.07 cm^2^ geometrical surface area) were used as the reference electrode, counter electrode and working electrode, respectively. To prepare the working electrode, as-synthesized samples are supported with carbon black (10 wt% for Pd cubes and PdAgPt HMNCs, 20 wt% for commercial Pt) by means of magnetic stirring and ultrasonic treatment. Then, the catalysts were dispersed in a mixture of isopropyl alcohol and Nafion solution (0.025 wt% Nafion dissolved in water/isopropyl alcohol, V/V = 9:1). After the GCE was cleaned and polished, 2 μL of the ink (about 360 μg mL^−1^ for Pd cubes and PdAgPt HMNCs, 720 μg mL^−1^ for commercial Pt) was dropped onto the GCE and dried in air. Before the electrocatalytic measurements, cyclic voltammetry (CV) cycles were conducted in N_2_-saturated HClO_4_ solution (0.1 mol L^−1^) from −0.21 to 0.9 V vs. SCE till the blank CVs stayed unchanged. Afterwards, the CV curves were recorded in N_2_-saturated HClO_4_ (0.1 mol L^−1^) and HCOOH (0.5 mol L^−1^) from −0.21 to 0.9 V at a sweep rate of 50 mV s^−1^ to estimate the activity of the electrocatalysts toward FAO. Accelerated durability tests were also carried out in the same electrolyte by repeated potential cycling between 0.3 V and 0.8 V vs. SCE at the sweep rate of 125 mV s^−1^. The CV curves after sweeping different cycles (from 100 to 30,000 cycles) were recorded in fresh electrolyte, N_2_-saturated HClO_4_ (0.1 mol L^−1^) and HCOOH (0.5 mol L^−1^), from −0.21 to 0.9 V at a sweep rate of 50 mV s^−1^.

## 3. Results and Discussion

### 3.1. From Pd Cubes to Pd@Ag Cubes

**Figure 1:**
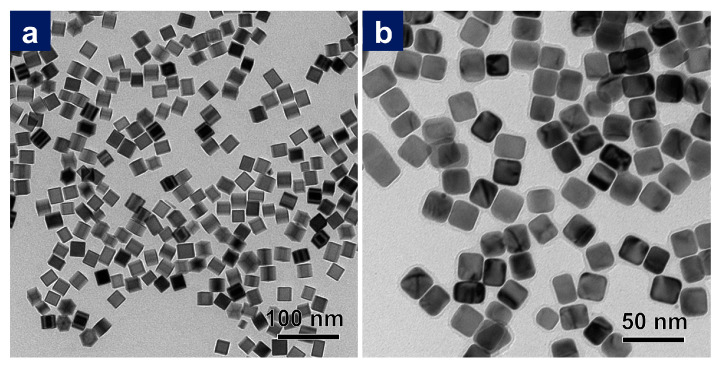
TEM images of (**a**) Pd cubes, and (**b**) Pd@Ag cube. Scale bar: as shown in figures.

The complete coverage of the silver shell on well-defined Pd cubes is an essential prerequisite for the formation of PdAgPt corner-satellite nanostructures. Figure 1 shows TEM images of Pd nanocubes and Pd@Ag nanocubes with well-controlled cubic morphologies and fairly close sizes (Figure S1a,b in Supplementary Materials). Due to a considerable lattice mismatch between Ag and Pd (4.8%), Moiré patterns could be clearly observed in the Pd region, which was a direct line of visual evidence for the epitaxial relationship between Pd cubes and Ag shells [48]. HRTEM images of a single Pd@Ag cube and the corresponding fast Fourier transform (FFT) image are displayed in Figure 3(a,a1). Lattice fringes with spacing of 1.93 Å and 2.03 Å in two directions could be indexed to the {200} plane of a face-centered cubic (fcc) lattice (1.945 Å for Pd {200}, 2.044 Å for Ag {200}). Accordingly, Pd@Ag nanocubes remained enclosed by six {100} facets. When focusing on the eight corners, it was not difficult to find the small expansion of {111} facets after forming the Ag shell, as a result of thermodynamic equilibrium. In general, the surface free energies for common low-index facets of fcc structures followed the order as {110} > {100} > {111}, and the final products were preferentially bounded by inactive facets to minimize surface free energy [49,50]. In this case, Ag atoms epitaxially grew from {100} facets of Pd cubes, thus altering the subsequent heterogeneous growth of Pt.

### 3.2. From Pd@Ag Cubes to PdAgPt HMNCs

**Figure 2:**
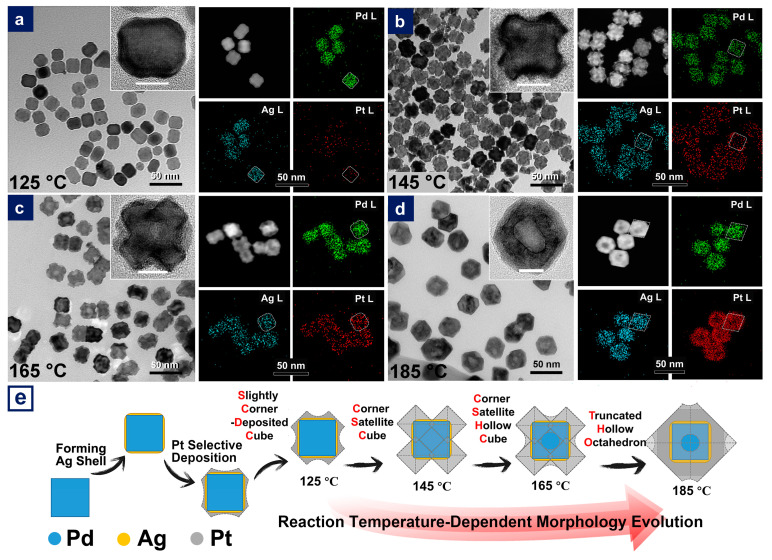
TEM images (scale bar: 50 nm) with high-resolution TEM (HRTEM) images of individual nanoparticles in the insets (scale bar in white: 10 nm), STEM images and corresponding energy disperse X-ray (EDX) mapping profiles (scale bar: 50 nm) of (**a**) slightly corner-deposited cubes (PdAgPt-SCDC, (**b**) corner-satellite cubes (PdAgPt-CSC), (**c**) corner-satellite hollow cubes (PdAgPt-CSHC) and (**d**) truncated hollow octahedra (PdAgPt-THO). (**e**) Schematic illustration of the morphological evolution of PdAgPt products.

**Figure 3:**
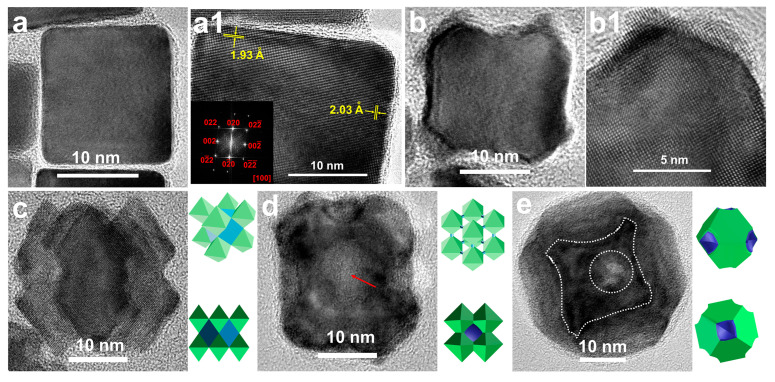
HRTEM images of (**a**,**a1**) Pd@Ag cubes with corresponding fast Fourier transform (FFT) image in the inset, (**b**,**b1**) PdAgPt-SCDC, (**c**) PdAgPt-CSC viewed from the <110> direction, (**d**)PdAgPt-CSHC from the <100> direction, and (**e**) PdAgPt-THO from the <100> direction. Scale bar: as shown in figures.

**Figure 4:**
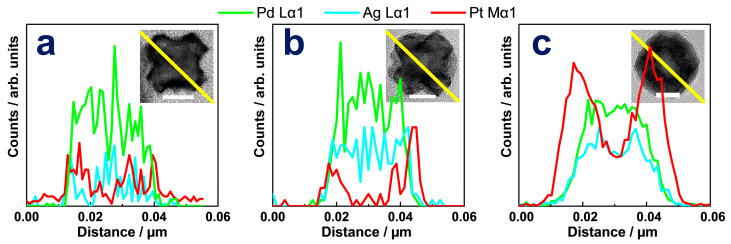
EDX line scanning profiles along the yellow line in the insets of (**a**) PdAgPt-CSC, (**b**) PdAgPt-CSHC, and (**c**) PdAgPt-THO. Scale bar in the insets: 10 nm.

The following step, namely epitaxial growth of Pt on Ag-remoulded surface, led to a series of trimetallic nanoparticles. Reaction temperature was a crucial factor for the morphological evolution of prepared PdAgPt nanocrystals. As seen in Figure 2, selective deposition of increasing Pt atoms led to the morphological evolution of as-obtained PdAgPt nanocrystals with the rise of the reaction temperature. At 125 °C, only a small number of Pt atoms were selectively deposited on the corners of nanocubes (Figure 2a and Figure 3(b,b1)). Consequently, the size of these slightly corner-deposited cubes (PdAgPt-SCDC) measured by the narrowest width through the particle center was nearly the same as that of Pd@Ag (Figure S1c). The satellite AgPt nanocrystals on eight corners of the central cubes, to some extent, became much more identifiable in products named as corner-satellite cubes (PdAgPt-CSC) at 145 °C (Figure 2b). HRTEM images of individual PdAgPt-CSC nanocrystals are viewed from the <100> (Figure 2b inset) and <110> (Figure 3c) directions. The morphologies of satellite nanocrystals were literally octahedral due to expanding {111} facets. Besides, dark-field scanning TEM (STEM) image and corresponding EDX elemental mapping profiles of PdAgPt-CSC are also presented in Figure 2b. Distribution of three metals met the properties of Pd@AgPt core-satellite nanostructures. When the reaction temperature rose above 165 °C, hollow side faces of central cubes and expanding {111} facets of satellite nanocrystals led to the formation of corner-satellite hollow cubes (PdAgPt-CSHC, Figure 2c and Figure 3d) and further truncated hollow octahedra (PdAgPt-THO, Figure 2d and Figure 3e). Combined with EDX line scanning profiles in Figure 4, Pt signals appeared to be stronger around the particle compared with that in central parts, while Pd and Ag signals were evenly distributed. It is also worth mentioning that the distribution of Pd in PdAgPt-THO was confined to a parallelogram instead of a square, which indicated the promoted diffusion of Pd element at 185 °C. As the morphologies of PdAgPt CSMNCs evolved, the particle size increased from 21.08 nm (PdAgPt-CSC) to 23.18 nm (PdAgPt-CSHC), and to 35.66 nm (PdAgPt-THO), showing a relatively narrow distribution as presented in Figure S1d–f.

**Figure 5:**
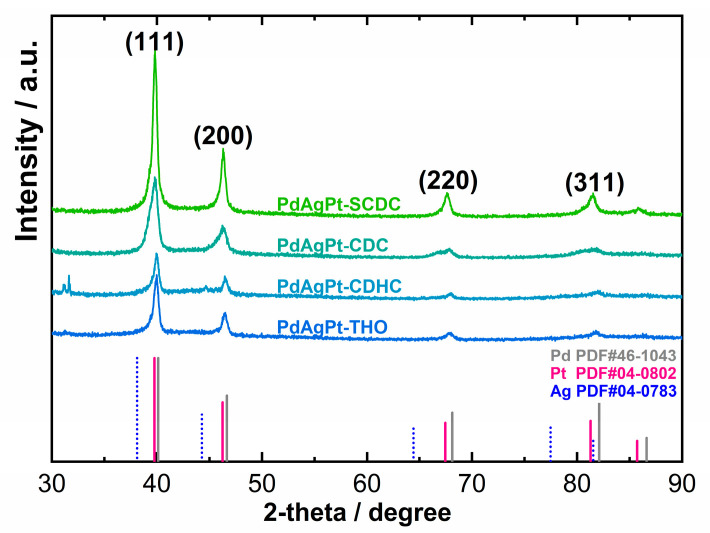
XRD patterns of four PdAgPt HMNCs.

**Figure 6:**
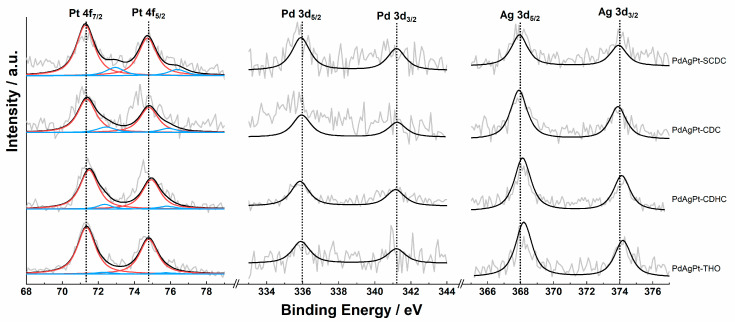
XPS spectra of Pt 4f, Pd 3d, and Ag 3d of the four PdAgPt HMNCs.

X-ray powder diffraction (XRD) patterns, as shown in Figure 5, further confirmed the fcc crystalline structures of all PdAgPt HMNCs. The diffraction peaks were located on the sites between pure Pd (PDF 46-1043) and Pt (PDF 04-0802), shifting to a larger angle than Ag (PDF 04-0783), which proved the two former metals were the major compositions. To a certain extent, the peak shifts in four XRD patterns were remarkably consistent with Vegard’s law. Despite the fact that XRD profiles reflected the alloy phase, the differences between atomic ratios detected by XPS and EDX confirmed the existence of heterogeneous nanostructures, as presented in Table 1. Because the XPS penetration depth was typically around 3 nm, the Pd 3d spectrum showed a doublet less clearly. On the whole, the chemical states of three metals in all PdAgPt existed predominantly as metallic states, as shown in Figure 6. The XPS binding energies of three metals are also listed in Table 1. Compared with the binding energies of the bulk metals (70.9 eV for Pt 4f_7/2_, 368.5 eV for Ag 3d_5/2_ [51] and 335.5 eV for Pd 3d_5/2_ [52]), the binding energies of Ag shifted lower and those of Pt and Pd higher for all PdAgPt HMNCs. These data suggested that the electronic structures were altered in PdAgPt HMNCs, specifically the electron densities of Pt were reduced.

### 3.3. The Growth Process of PdAgPt-THO

**Figure 7:**
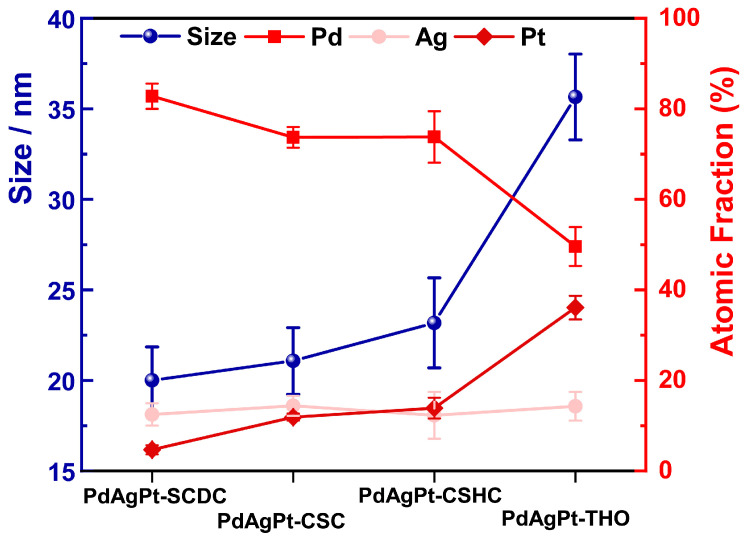
Evolution for the particle sizes and compositions evaluated by EDX in the four types of PdAgPt HMNCs.

**Figure 8:**
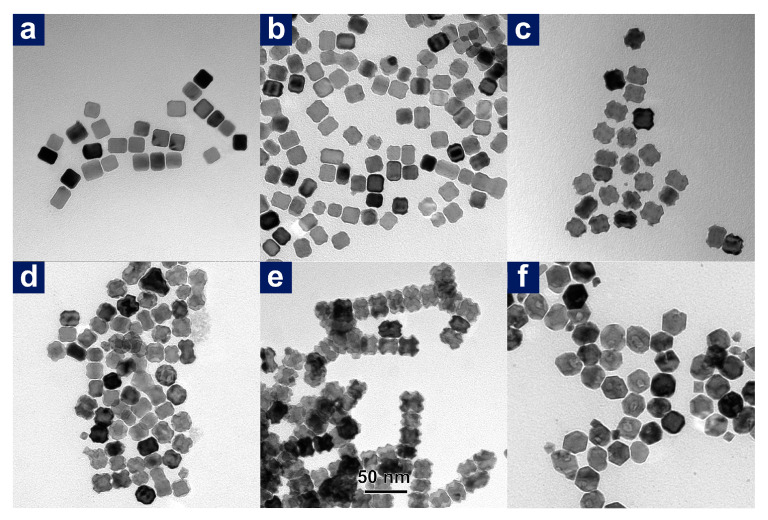
TEM images of intermediate products at different reaction stages to form PdAgPt-THO at the reaction temperature of 185 °C: (**a**) 5 min, (**b**) 10 min, (**c**) 30 min, (**d**) 1 h, (**e**) 2 h and (**f**) 3 h. Scale bar: 50 nm.

As Figure 2e outlines the morphological evolution of PdAgPt HMNCs in a systematical way, more Pt atoms were selectively deposited on the corners of the central cubes, thus causing expanding {111} facets, and the shape developed into truncated octahedra with the rise of the reaction temperature from 125 °C to 185 °C. Figure 7 presents the evolution for the particle sizes and compositions detected by EDX. The gradual rise in Pt proportion together with the decline of Pd atomic fraction revealed that the promoted epitaxial growth of Pt at higher temperatures was the primary reason for greater particle sizes. To elucidate the growth process of truncated octahedra, time-dependent TEM images at the reaction temperature of 185 °C are shown in Figure 8. In the initial stage (reaction time, *t* = 5 min to 10 min), the morphology of the Pd@Ag went unchanged until enough deposition of Pt atoms occurred at the cube corners selectively. When *t* =30 min, a quite number of Pt atoms grew epitaxially on the surface of Pd@Ag and formed relatively well-defined {111} facets with Ag, the product appeared to take on an intermediate shape between PdAgPt-SCDC and PdAgPt-CSC (Figure 8c). After the reaction proceeded to *t* = 1 h, galvanic displacement between the Pt precursor and the bimetallic inner core (Ag and Pd) began to play a much larger role in shaping the final product. In fact, the hollow side faces formed at high temperatures were most probably caused by galvanic displacement between Pt precursors and the Pd core [53]. High reaction temperatures facilitated not only the deposition of Pt but also the diffusion of metal atoms. Only when the inside-out diffusion of Pd occurred could galvanic displacement help achieve cavities on the side faces of central cubes. Hence, when the reaction time *t* = 2 h, hollow nanostructures emerged with the further growth of corner satellites, as shown in Figure 8e. In the last stage of PdAgPt-THO formation (*t* = 3 h), corner-satellite heterogeneous nanocrystals evolved into truncated octahedral nanostructures, which were mainly composed of eight {111} facets, and with random cavities on {100} (Figure 8f). Taken together, the reaction time and the reaction temperature, as two common determining factors, were vital to controlling the extent of reaction and the final morphology of prepared PdAgPt products in this seed-mediated growth process.

### 3.4. The Roles of Ag and Bromide Ions

The morphology of corner-satellite nanocrystals was predominantly controlled by growth kinetics of Pt atoms with the aid of Ag. When K_2_PtCl_6_ was applied as a Pt precursor and its concentration was modulated using a syringe pump, it was hard to observe the formation of clear {111} facets on the corners of central cubes (Figure S2). With the slow injection rate of Pt salts, the Stranski–Krastanov growth mode was preferred instead of the full-island growth mode, which made it difficult to form corner-satellite nanocrystals even with the further deposition of Pt (Figure S3). To some extent, Pt acetylacetonate as a Pt source instead of K_2_PtCl_6_ was conducive to well-defined shapes, because it could be less easily reduced. While heating at 145 °C for 30 min to form Ag shells, glycolaldehyde is acknowledged as a primary reductant in polyol [54], which also plays a part in the formation of hollow structures [55]. By means of the galvanic displacement between Pt precursors and Ag atoms, the Ag shell served as a promotive masking in the reduction and deposition of Pt, and the Ag^+^ ion released by displacement would be quickly reduced in the polyol process. As demonstrated in Figure S4, the deposition of Pt atoms from Pt(acac)_2_ upon Pd nanocubes could be hardly observed at 105 °C or 145 °C, while more self-nucleation of Pt emerged at 185 °C. The results, combined with Figure S5, the comparison of products with and without Ag, confirmed that the existence of complete and continuous Ag shells contributed immensely to the reduction and selective deposition of Pt. Obviously, an incomplete coverage of Ag made it difficult to form well-defined AgPt corner-satellite structures (Figure S6), while excessive Ag caused particle agglomeration (Figure S7). With Ag shells, the concaved side faces of PdAgPt occurred at high reaction temperatures, when the diffusion and deposition were both thermally promoted.

**Figure 9:**
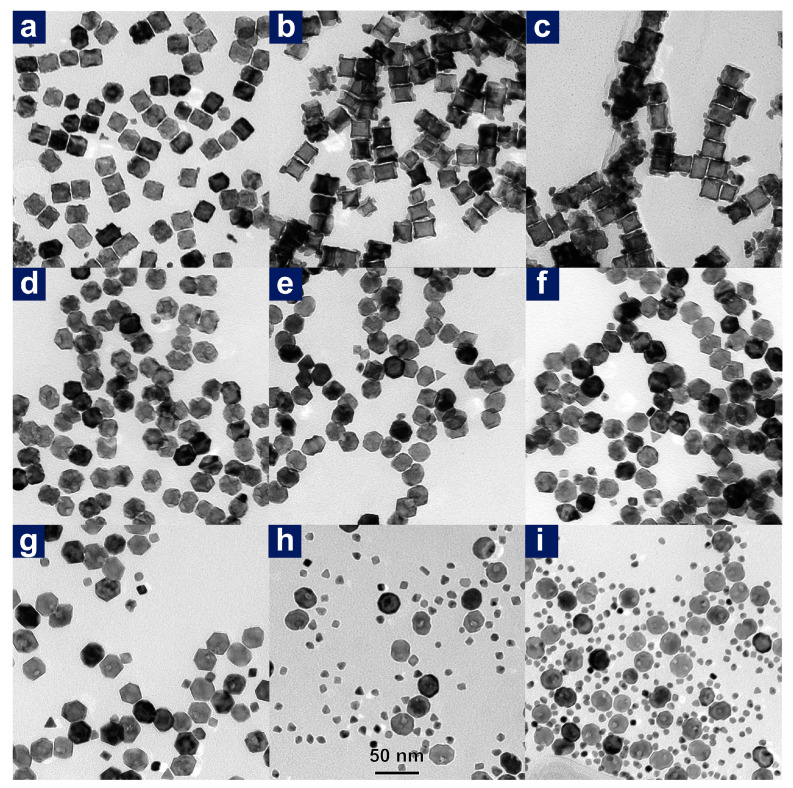
TEM images of PdAgPt products obtained without adding any KBr at (**a**) 145 °C, (**b**) 165 °C, and (**c**) 185 °C; and PdAgPt products prepared at 185 °C with different KBr concentrations of (**d**) 0.8 mmol L^−1^, (**e**) 4.2 mmol L^−1^, (**f**) 8.3 mmol L^−1^, (**g**) 16.7 mmol L^−1^, (**h**) 50 mmol L^−1^ and (**i**) 250 mmol L^−1^. Scale bar: 50 nm.

Unlike the Ag shell acting as a promotive masking for the formation of corner-satellite structures, halide ions, especially bromide ions, had more complex effects on the preparation of PdAgPt CSMNCs. On the one hand, both Pt precursors and released Ag^+^ or Pd^2+^ could interact with the bromide ions in solution, leading to altered reduction potential, and thus promoted galvanic displacement. On the other hand, highly selective adsorption of Br^−^ on the {100} facets allowed the geometrical anisotropy of the central cubes to direct the localized deposition of satellite AgPt nanocrystals along with localized cavities at high temperatures. In a standard procedure, a certain concentration of KBr (13.4 mmol L^−1^) was added before the reduction of Ag. However, without the acceleration of Br^−^ ions, Pt atoms derived from Ag-promoted reduction were not sufficient for the growth of AgPt satellites on the corners. As demonstrated in Figure 9a–c, more heterogenous particles adhered to PdAgPt nanocrystals synthesized without Br^−^, and the corners became more protruding when the temperature rose from 145 °C to 185 °C. As the concentration of KBr increased from 0.8 to 8.3 mmol L^−1^, overall morphologies of most PdAgPt nanocrystals prepared at 185 °C remained truncated octahedra with minor cavities on the surfaces, as seen in Figure 9d–f. Figure 9g–i shows the PdAgPt nanocrystals prepared at 185 °C with an excessively high concentration of KBr (16.7 mmol L^−1^, 50 mmol L^−1^, 250 mmol L^−1^). Concaved ellipsoidal particles rather than truncated octahedra became the main products due to the favoured exposure of {100} facets promoted by excess Br^−^. Moreover, with the increasing concentration of KBr, a rising number of small alloy particles were generated in the polyol because of excessively facilitated galvanic displacement. Since the chemisorption ability of halide ions increased in the order of Cl <Br < I [55], Cl^−^ ions were inefficient for promoting the growth of Pt atoms and guiding the evolution of satellites exclusively on the corners. When moderate concentration of KCl (12.5 mmol L^−1^) was applied as a capping agent instead of KBr, PdAgPt nanocrystals acquired at 145 °C only presented relatively selective deposition on the corners. In this case, less-facilitated reduction of Pt was not sufficient for shaping well-defined corner satellites (Figure S8a). At 185 °C, as galvanic displacement between Pd and Pt precursors hardly occurred with non-existent KBr and sufficient KCl (67 mmol L^−1^), prepared PdAgPt truncated octahedral nanocrystals were predominantly bounded by {111} facets without hollow interiors (Figure S8b). In contrast, when sufficient KI (67 mmol L^−1^) instead of KBr was added, the highly boosted galvanic displacement between Pd and Pt precursors dissolved the original cubic morphology at 85 °C (Figure S8c). Therefore, a moderate concentration of Br^−^ played a crucial role in the evolution of corner-satellite nanostructures by accelerating the anisotropic growth of Pt, and simultaneously, the formation of hollow side faces by properly enhanced galvanic displacement.

### 3.5. Structure-Related Electrocatalytic Properties

**Figure 10:**
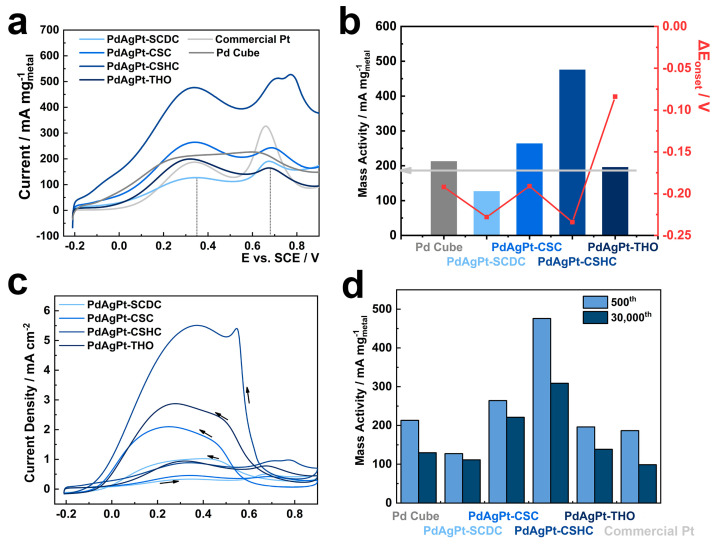
Electrochemical performances of as-prepared PdAgPt HMNCs compared with Pd cubes and commercial Pt. (**a**) Mass-normalized CV curves in formic acid (0.5 mol L^−1^) and HClO_4_ (0.1 mol L^−1^), (**b**) onset potential shift values toward that of commercial Pt, and mass activities of I_a_ at 0.35 V (vs. saturated calomel electrode (SCE)), light grey arrow line represent the level of commercial Pt, (**c**) ECSA-normalized CV curves of as-prepared PdAgPt HMNCs, (**d**) mass activities of I_a_ in the 500th and 30,000th cycle.

As a structure-sensitive electrocatalytic reaction, FAO was chosen to assess the electrochemical performance of as-prepared PdAgPt HMNCs, benchmarked against the original Pd cubes and a commercial Pt catalyst (average size: 10 nm). The specific electrochemically active surface areas (ECSAs) of PdAgPt HMNCs were estimated by accumulating the charge of the hydrogen adsorption/desorption area in the cyclic voltammetry (CV) curves obtained in N_2_-saturated HClO_4_ solution (0.1 mol L^−1^) (Figure S9). While the ECSA of the largest PdAgPt-THO was only 211.48 cm^2^ mg^−1^, around half of that of commercial Pt (437.15 cm^2^ mg^−1^), the value for PdAgPt-CSC and PdAgPt-CSHC reached 579.89 cm^2^ mg^−1^ and 538.63 cm^2^ mg^−1^, indicating the superiority of CSMNCS. To investigate the electrocatalytic properties of PdAgPt HMNCs, the metal mass-normalized CV curves toward FAO are given in Figure 10a, and part of the ECSA-normalized CV curves are shown in Figure 10c. There were generally two apparent peaks during the forward scan, which could refer to the direct oxidation pathway (FAO pathway a) and the indirect oxidation pathway (FAO pathway b), respectively [13,14,19,39,56]. Therefore, the current ratio between peak I_a_ and peak I_b_ is often employed to assess the dominant FAO pathway [57,58,59]. As depicted in Figure 10a, the two anodic peaks were centered at 0.349 V and 0.677 V for the commercial Pt, while the FAO on the Pd cubes presented one broad peak centered at about 0.25 V. During the 30,000 cycles of accelerated durability tests for FAO, the precise peak center positions of two FAO oxidation pathways for all electrocatalysts are listed in Table S1. Taking into consideration all the factors, the current densities at 0.35 V vs. SCE were applied as I_a_, and current densities at 0.68 V vs. SCE were used as I_b_ for better comparison. The mass activities (I_a_) in the direct oxidation pathway for all samples are shown in Figure 10b. Surprisingly, PdAgPt multi-metal nanocrystals with corner-satellite structures exhibited higher mass activities than Pd cubes (213.11 mA mg^−1^) after 500 accelerated cycles of FAO. The mass activities of PdAgPt-CSHC (475.99 mA mg^−1^) and PdAgPt-CSC (264.16 mA mg^−1^) were about 2.55 times and 1.42 times as large as that of commercial Pt (186.42 mA mg^−1^). Compared with other noble metal-based electrocatalyst reported before, the PdAgPt-CSHC also showed noteworthy superiority in direct FAO mass activity (Table S2). It verified the obviously enhanced catalytic activity of PdAgPt HMNCs with both corner satellites and hollow structures. The current ratios of I_a_ to I_b_ (1.09 for PdAgPt-CSC, 0.96 for PdAgPt-CSHC, and 0.60 for commercial Pt), in this condition displayed that the direct formic acid oxidation pathway of PdAgPt CSMNCs was highly promoted. Moreover, the onset oxidation potential of the FAO, E_onset_, defined as the potential at which 10% of the I_a_ is reached, could also be used to evaluate the electrocatalytic performance. The shift values from the onset potential of commercial Pt, i.e., ΔE_onset_, are also depicted in Figure 10b. The onset potentials of all the PdAgPt HMNCs except PdAgPt-THO were close to that of Pd cubes, about 0.20 V negative to the E_onset_ of commercial Pt. Hence, it can be inferred that the special corner-satellite structures owed better electrocatalytic properties than normal heterogeneous structure. The reasons for the improvement of PdAgPt-CSHC sample are listed as follows. (i) The “third-body” effect: the indirect oxidation pathway preferred by Pt was suppressed, since active sites on the surface of corner satellites were reduced due to geometrical hindrance [27,60]. (ii) The electronic effect: because the electronic structure of Pt was altered in the heterogeneous nanostructure, the adsorption of HCOO* species was promoted, and the direct oxidation pathway of FAO was enhanced [9,16]. (iii) The synergetic effect: with the aid of Pd and Ag, Pt atoms showed enhanced catalytic activity toward FAO with higher proportion of the direct oxidation pathway, especially in hollow PdAgPt HMNCs.

**Figure 11:**
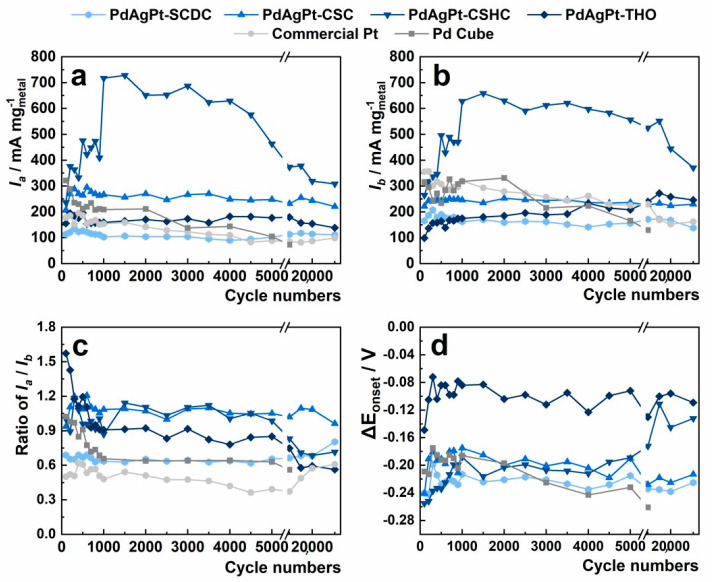
(**a**) Mass activities I_a_ at 0.35 V vs. SCE, (**b**) mass activities I_b_ at 0.68 V vs. SCE, (**c**)current ratios of I_a_ to I_b_, and (**d**) onset potential shift values toward that of commercial Pt, ΔE_onset_, for all electrocatalysts in the whole 30,000 cycles of FAO.

Heterogeneous shell of AgPt enormously improved the durability of whole nanocrystal in electrocatalysis. The electrocatalytic properties of all samples during 30,000 cycles of accelerated durability tests for FAO are shown in Figure 11. There is no doubt that the mass activities of both Pd cubes and commercial Pt dropped off markedly and steadily, during 30,000 cycles of accelerated CV measurements (Figure 10d). The retained mass activity I_a_ for commercial Pt was 98.67 mA mg^−1^, which was 53% of I_a_ in the 500th. As for PdAgPt HMNCs, from the analysis of electrocatalytic performance during 30,000 cycles, it may be inferred that the structures of PdAgPt-SCDC and PdAgPt-CSC were almost unchanged. The mass activity of PdAgPt-SCDC remained constantly low in the whole durability test, despite the fact that its onset potential kept close to that of Pd cubes. As well-defined AgPt corner satellites took shape in PdAgPt-CSC, the mass activity I_a_ after 30,000 cycles still reached 220.96 mA mg^−1^ (84%), showing greatly enhanced durability. The combination of corner satellites and hollow nanostructures in PdAgPt-CSHC achieved the highest direct FAO activity after 1000 cycles, which may have resulted from increasing active sites with the gradual loss of Ag. The high performance of PdAgPt-CSHC fell into decay after 10,000 cycles, the onset potential became less negative and the current ratio of I_a_ to I_b_ also descended from 0.9~1.2 to below 0.8 (Figure 11c,d). Still, its mass activity at 0.35 V after 30,000 cycles (308.78 mA mg^−1^) maintained 65% of the mass activity in the 500th cycle, about 3.13-fold of that of commercial Pt. One possible explanation is that the deformation of hollow nanostructures took place after the dissolution of Ag in the heterogeneous shell, as Ag resisted the dissolution of Pd/Pt. Moreover, Pt alloyed with Ag has been proved to exhibit higher tolerance to CO poisoning, which increases the CO resistance and thus durability of nanocatalysts [14,61]. After the dissolution of Ag, the electrocatalytic performance decay of PdAgPt samples was inevitable. Similar decay in electrocatalytic performance occurred in the other hollow structure (PdAgPt-THO), whose retained mass activity after 30,000 cycles amounted to 138.49 mA mg^−1^ (71%). Overall, PdAgPt HMNCs displayed better FAO durability than the original Pd cubes and commercial Pt. Therefore, by constructing the corner satellites with sacrificial Ag on sheltered Pd cores, Pt atoms fulfilled their essential role, and all precious metals could achieve higher utilization efficiency in a long-term way.

## 4. Conclusions

In summary, we have presented a facile but effective strategy to prepare PdAgPt HMNCs with corner-satellite nanostructures. The morphologies, compositions and sizes of as-prepared HMNCs are well controlled in the seed-mediated evolution process from Pd@Ag cubes into final truncated hollow octahedra (PdAgPt-THO). Selective deposition of Pt, and further growth of AgPt corner satellites are contingent upon two decisive factors: (I) promotive masking of Ag shells, (II) geometrical anisotropy of the central cubes with the help of Br^−^ in moderate concentration. Furthermore, the galvanic displacement promoted at high temperatures also plays a key role in the formation of localized hollow structures. By utilizing the “third-body” effect, the electronic effect and the synergetic effect, PdAgPt CSMNCs, especially PdAgPt-CSHC with a hollow structure, exhibit remarkably enhanced activity toward FAO in direct oxidation pathway, with more negative onset potential compared with that of commercial Pt. By virtue of the heterogeneous shell composed of Ag and Pt, the long-term durability of PdAgPt HMNCs has been improved tremendously. Hopefully, this work can pave the way for a more insightful understanding of the relationship between the structure and the function of HMNCs.

## Figures and Tables

**Table 1:** Surface compositions of the metals determined by X-ray photoelectron spectra (XPS) measurements compared with EDX analyses and observed XPS binding energies.

	XPS	EDX	Pt 4f7/2	Ag 3d5/2	Pd 3d5/2
PdAgPt-SCDC	Pd_32_Ag_44_Pt_24_	Pd_83_Ag_12_Pt_5_	71.28	367.93	335.90
PdAgPt-CSC	Pd_29_Ag_37_Pt_34_	Pd_74_Ag_14_Pt_12_	71.35	367.89	335.94
PdAgPt-CSHC	Pd_36_Ag_31_Pt_33_	Pd_74_Ag_12_Pt_14_	71.48	368.12	335.85
PdAgPt-THO	Pd_21_Ag_28_Pt_51_	Pd_50_Ag_14_Pt_36_	71.34	368.19	335.89

## Supplementary Materials

The following are available online at https://www.mdpi.com/2079-4991/11/2/340/s1, Figure S1: Size distribution histograms of (a) Pd cubes, (b) Pd@Ag, (c) PdAgPt-SCDC, (d) PdAgPt-CSC, (e) PdAgPt-CSHC, and (f) PdAgPt-THO. Figure S2: TEM images of PdAgPt nanoparticles applying K_2_PtCl_6_ as Pt precursors instead of Pt(acac)_2_ by modulating injection rate via syringe pump (Pt element, 4.8 mmol L^−1^): injected in (a) 1 min, (b) 1 h, (c) 2.5 h, and (d) 5 h (reaction time prolonged by another two hours). Figure S3: TEM image of PdAgPt nanoparticles applying K_2_PtCl_6_ as Pt precursors instead of Pt(acac)_2_ (Pt element, 9.6 mmol L^−1^). Figure S4: TEM images of PdPt nanoparticles without Ag shells, applying Pt(acac)_2_ as Pt precursors, and the reaction temperatures are set as (a) 105 °C, (b) 145 °C, and (c) 185 °C. Figure S5: TEM images of (a,b) PdPt without Ag shells and (c,d) PdAgPt with preformed Ag shells when applying K_2_PtCl_6_ as Pt precursors, the reaction temperatures are set as (a,c) 105 °C and (b,d) 125 °C. Figure S6: TEM images of PdAgPt nanoparticles, applying Pt(acac)_2_ as Pt precursors, with (a) only one fourth and (b,c) half amount of Ag, the reaction temperatures are set as (a,b) 145 °C and (c) 185 °C. Figure S7: TEM image of Pd@Ag nanoparticles with double amount of Ag. Figure S8: TEM images of PdAgPt nanoparticles, applying KCl instead of KBr at (a) 145 °C (12.5 mmol L^−1^), and (b) 185 °C (67 mmol L^−1^); (c) applying KI (67 mmol L^−1^) instead of KBr at 85 °C. Figure S9: (a−e) CV curves in N_2_-saturated HClO_4_ solution at a sweep rate of 50 mV s^−1^ from −0.21 to 0.9 mV (vs. SCE) and (f) calculated ECSAs for carbon supported commercial Pt particles and as-prepared PdAgPt samples. Table S1: The peak center positions (V, vs. SCE) of the direct oxidation pathway (I_a_) and the indirect oxidation pathway (I_b_) for Pd cubes, commercial Pt and four PdAgPt HMNCs in 30,000 cycles of FAO. Table S2: Comparison of the direct FAO mass activity of the PdAgPt CSMNCs with other reported catalysts.
