# Supplementary material for: PdAgPt Corner-Satellite Nanocrystals in Well-Controlled Morphologies and the Structure-Related Electrocatalytic Properties
Source: Nanomaterials (Basel). 2021 Jan 29;11(2):340. doi: 10.3390/nano11020340 (PMC7911664; doi:10.3390/nano11020340)
Supplement: Supplementary file 1 [file nanomaterials-11-00340-s001.pdf]

Supplementary Materials

PdAgPt Corner-Satellite Nanocrystals in Well-Controlled Morphologies and the Structure-Related Electrocatalytic Properties

Hehe Qian, Jianzhou Wu, Yongsheng Guo and Wenjun Fang*

Department of Chemistry, Zhejiang University, Hangzhou 310058, China

*Corresponding Author. E-mail Address: fwjun@zju.edu.cn. Tel: +86-571-88981416. Fax: +86-571-88981416.

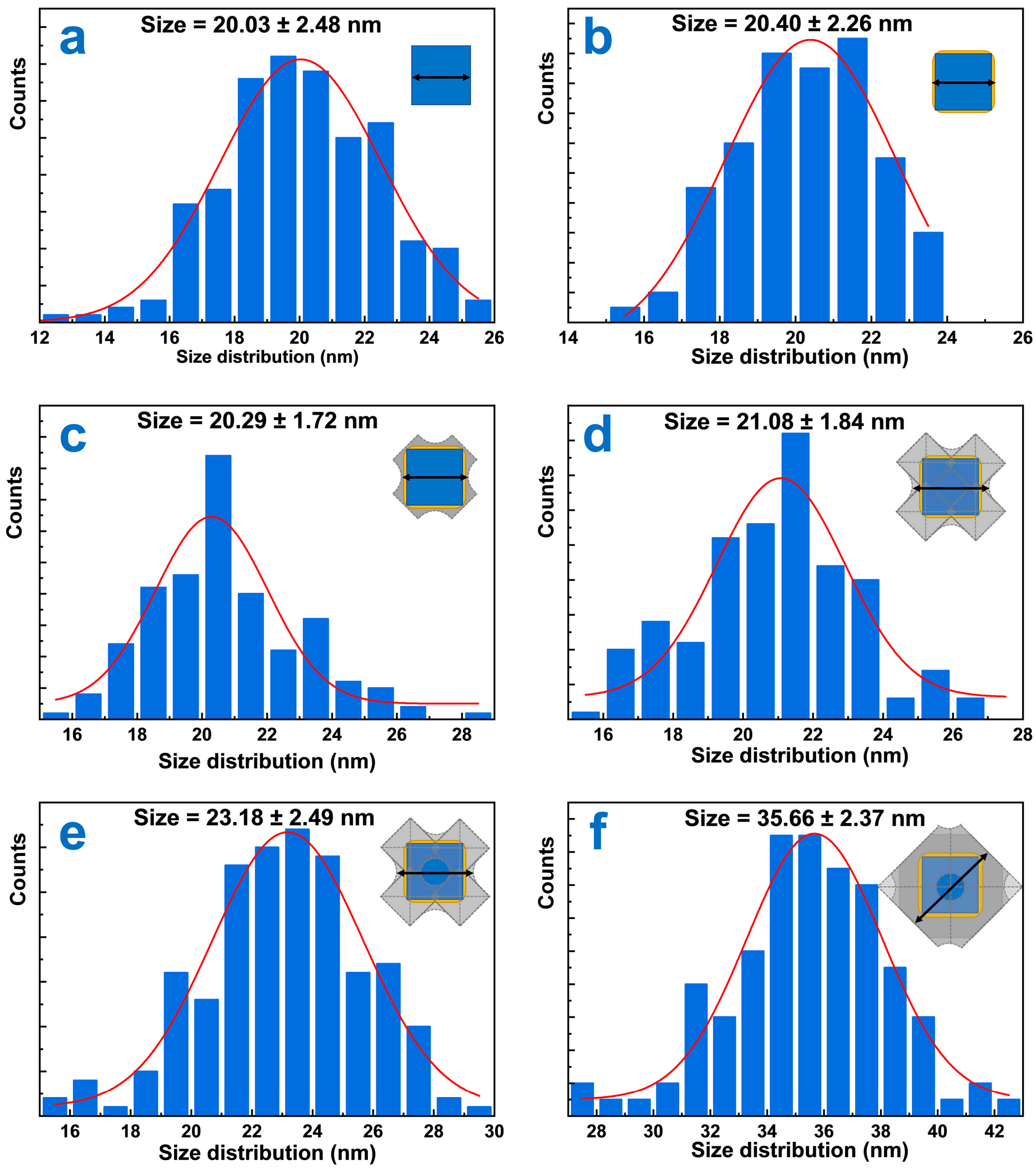

Figure S1. Size distribution histograms of (a) Pd cubes, (b) Pd@Ag, (c) PdAgPt-SCDC, (d) PdAgPt-CSC, (e) PdAgPt-CSHC, and (f) PdAgPt-THO.

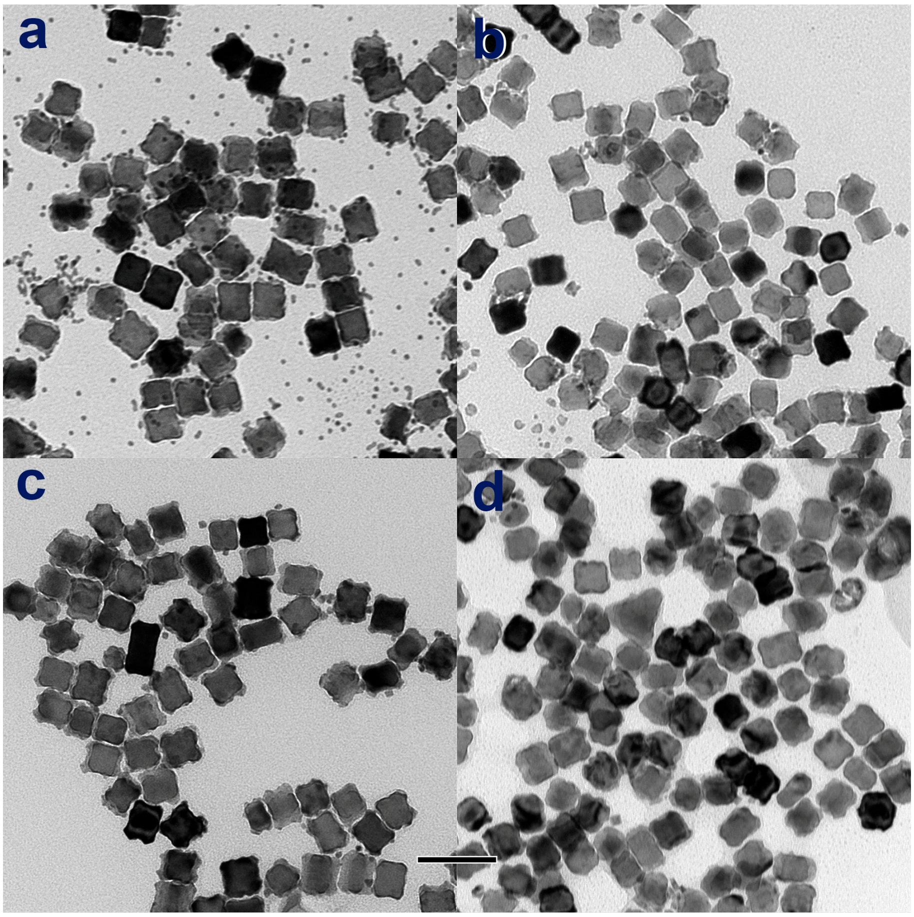

Figure S2. TEM images of PdAgPt nanoparticles applying K_2PtCl_6 as Pt precursors instead of $Pt(acac)_2$ by modulating injection rate via syringe pump (Pt element, 4.8 mmol L^{-1}): injected in (a) 1 min, (b) 1 h, (c) 2.5 h, and (d) 5 h (reaction time prolonged by another two hours). Scale bar: 50 nm.

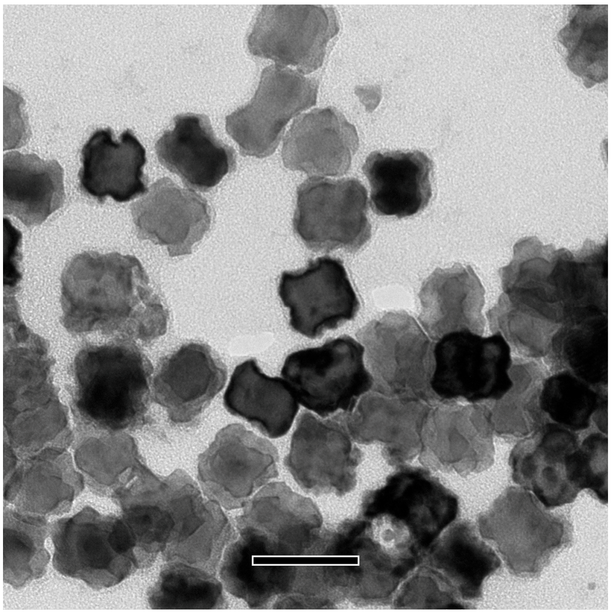

Figure S3. TEM image of PdAgPt nanoparticles applying K_2PtCl_6 as Pt precursors instead of $Pt(acac)_2$ (Pt element, 9.6 mmol L^{-1}). Scale bar: 50 nm.

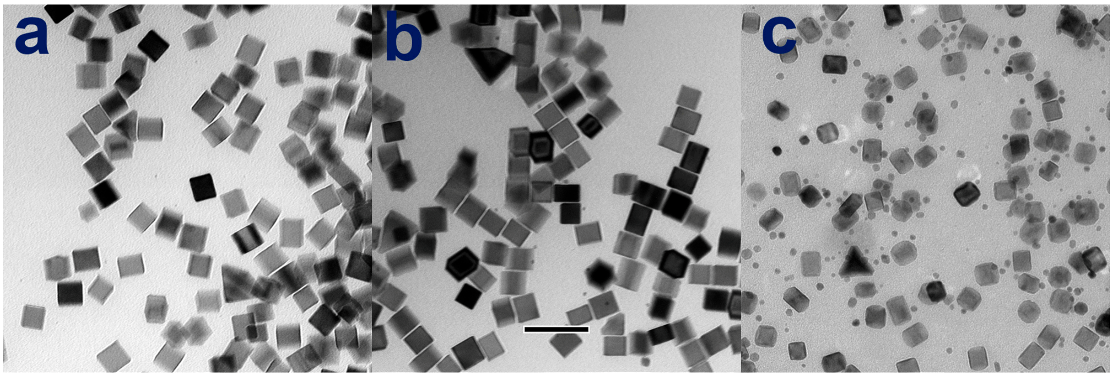

Figure S4. TEM images of PdPt nanoparticles without Ag shells, applying $Pt(acac)_2$ as Pt precursors, and the reaction temperatures are set as (a) 105 °C, (b) 145 °C, and (c) 185 °C. Scale bar: 50 nm.

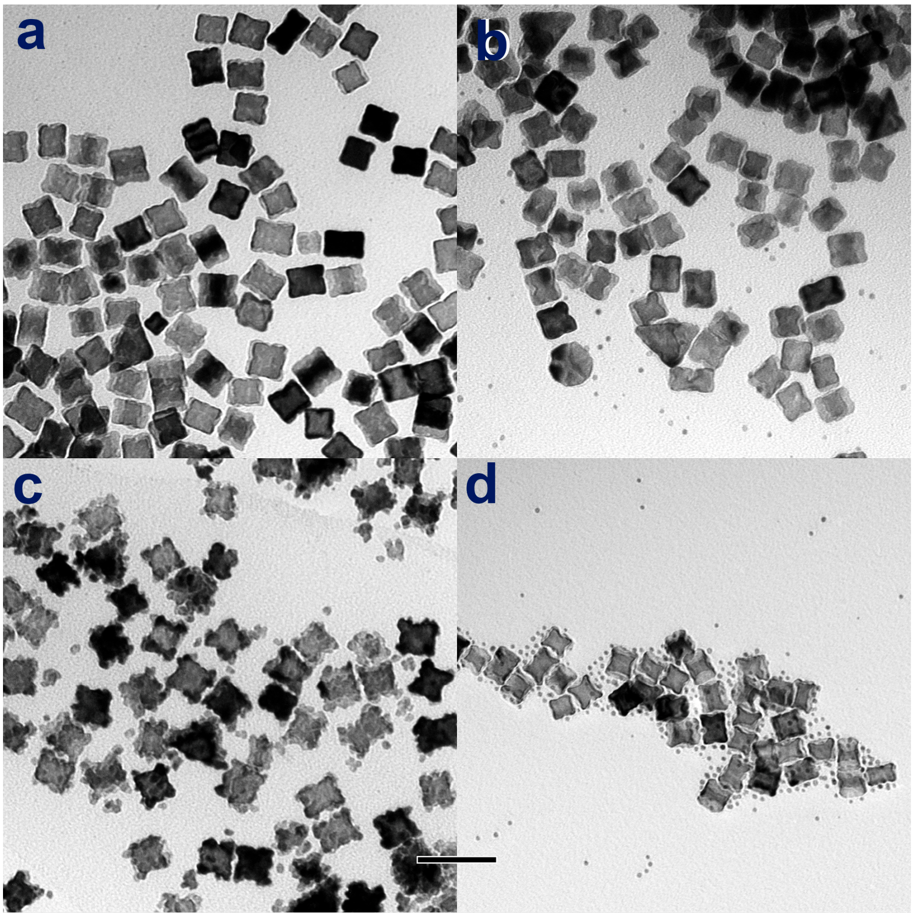

Figure S5. TEM images of (a,b) PdPt without Ag shells and (c,d) PdAgPt with preformed Ag shells when applying K_2PtCl_6 as Pt precursors, the reaction temperatures are set as (a,c) 105 °C and (b,d) 125 °C. Scale bar: 50 nm.

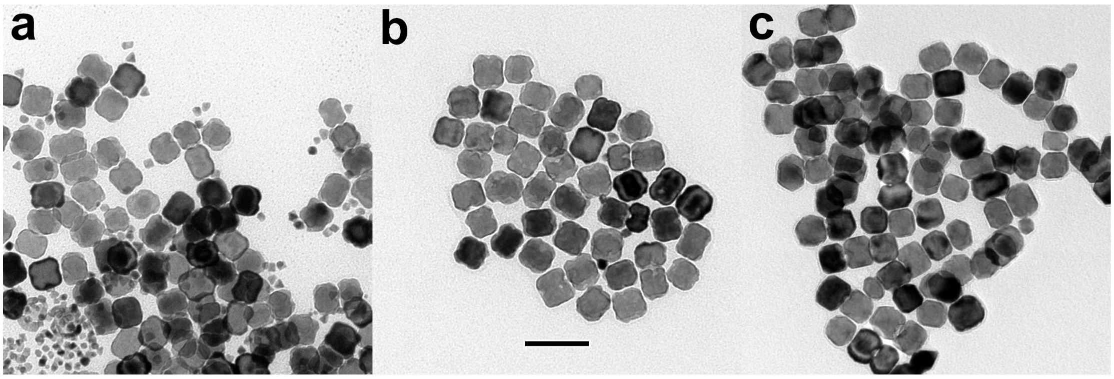

Figure S6. TEM images of PdAgPt nanoparticles, applying $Pt(acac)_2$ as Pt precursors, with (a) only one fourth and (b,c) half amount of Ag, the reaction temperatures are set as (a,b) 145 °C and (c) 185 °C. Scale bar: 50 nm.

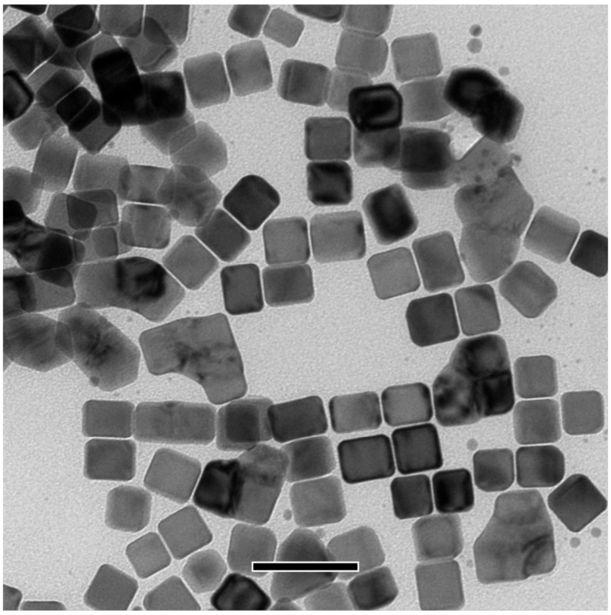

Figure S7. TEM image of Pd@Ag nanoparticles with double amount of Ag. Scale bar: 50 nm.

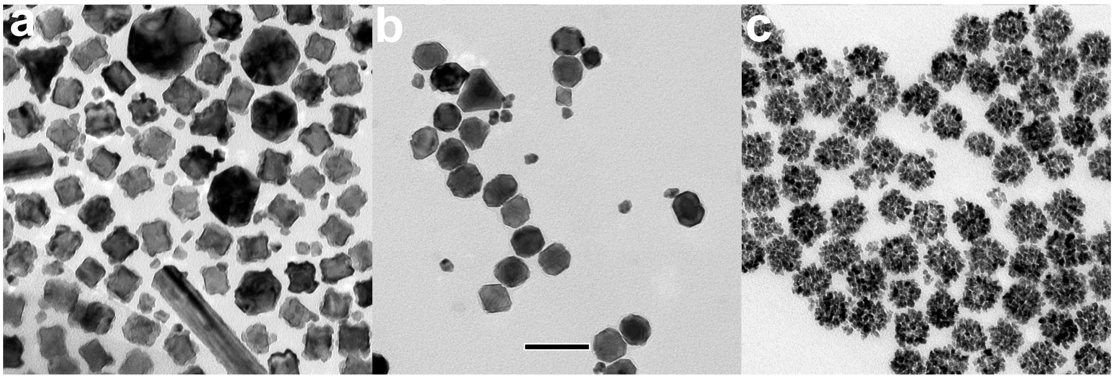

Figure S8. TEM images of PdAgPt nanoparticles, applying KCl instead of KBr at (a) 145 °C (12.5 mmol L^{-1}), and (b) 185 °C (67 mmol L^{-1}); (c) applying KI (67 mmol L^{-1}) instead of KBr at 85 °C. Scale bar: 50 nm.

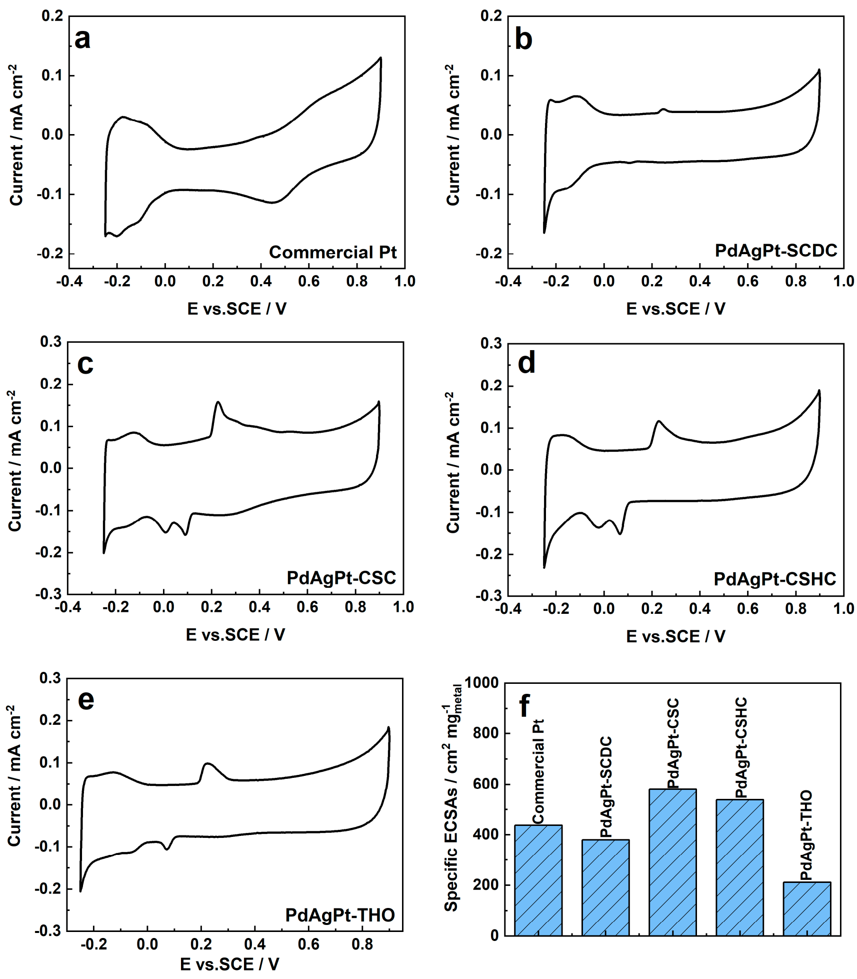

Figure S9. (a−e) CV curves in N_2-saturated $HClO_4$ solution at a sweep rate of 50 mV s^{-1} from −0.21 to 0.9 mV (vs. SCE) and (f) calculated ECSAs for carbon supported commercial Pt particles and as-prepared PdAgPt samples.

Table S1. The peak center positions (V, vs. SCE) of the direct oxidation pathway (I_a) and the indirect oxidation pathway (I_b) for Pd cubes, commercial Pt and four PdAgPt HMNCs in 30,000 cycles of FAO.

	Pd cube		***PdAgPt-SCDC***		***PdAgPt-CSC***		***PdAgPt-CSHC***		***PdAgPt-THO***		***Commercial Pt***	
	**I_a**	**I_b**	**I_a**	**I_b**	**I_a**	**I_b**	**I_a**	**I_b**	**I_a**	**I_b**	**I_a**	**I_b**
100th	/	0.6	0.391	0.708	0.385	0.701	0.39	/	0.334	/	0.348	0.67
200th	/	0.601	0.391	0.697	0.357	0.687	0.414	/	0.334	/	0.358	0.668
300th	0.231	/	0.383	0.689	0.342	0.678	0.349	/	0.336	0.681	0.342	0.665
400th	0.232	/	0.343	0.674	0.345	0.692	0.364	/	0.334	0.678	0.342	0.661
500th	0.224	/	0.349	0.677	0.349	0.684	0.351	/	0.324	0.677	0.343	0.661
600th	/	/	0.357	0.675	0.336	0.678	0.342	/	0.345	0.703	0.339	0.662
700th	/	/	0.351	0.67	0.337	0.688	0.344	/	0.32	0.678	0.332	0.655
800th	/	/	0.355	0.672	0.335	0.684	0.351	/	0.308	0.675	0.335	0.652
900th	/	/	0.36	0.67	0.344	0.689	0.345	/	0.319	0.678	0.345	0.651
1000th	/	/	0.359	0.67	0.339	0.685	0.359	/	0.312	0.676	0.322	0.651
1500th	/	/	0.359	0.673	0.336	0.684	0.329	0.701	0.308	0.675	0.331	0.65
2000th	/	/	0.349	0.668	0.339	0.683	0.337	0.707	0.32	0.681	0.326	0.649
2500th	/	/	0.363	0.673	0.348	0.689	0.339	0.706	0.319	0.678	0.322	0.65
3000th	/	/	0.359	0.672	0.337	0.683	0.338	0.707	0.319	0.678	0.326	0.649
3500th	/	/	0.351	0.671	0.334	0.678	0.345	0.71	0.334	0.687	0.326	0.645
4000th	/	/	0.359	0.676	0.338	0.684	0.345	0.71	0.319	0.681	0.345	0.646
4500th	/	/	0.363	0.673	0.339	0.684	0.344	0.704	0.318	0.68	0.326	0.646
5000th	/	/	0.356	0.678	0.334	0.684	0.338	0.703	0.316	0.684	0.324	0.645
10000th	/	/	0.352	0.675	0.345	0.687	0.342	0.7	0.331	0.695	0.358	0.649
15000th	/	/	0.351	0.676	0.334	0.683	0.359	0.703	0.35	0.684	0.375	0.657

20000th	/	/	**0.345**	**0.677**	**0.336**	**0.684**	**0.345**	**0.695**	**0.345**	**0.683**	**0.377**	**0.657**
30000th	/	/	**0.357**	**0.675**	**0.348**	**0.684**	**0.37**	**0.702**	**0.38**	**0.684**	**0.369**	**0.657**
Average	/	/	**0.359**	**0.677**	**0.342**	**0.685**	**0.351**	**0.704**	**0.328**	**0.681**	**0.341**	**0.654**

Table S2. Comparison of the direct FAO mass activity of the PdAgPt CSMNCs with other reported catalysts.

Samples	Electrolyte	Mass activity (mA mg^{-1})	Normalized by	References
PdAgPt-CSC	0.1 M $HClO_4$+0.5 M HCOOH	264.16	Metal mass	This work
PdAgPt-CSHC	0.1 M $HClO_4$+0.5 M HCOOH	475.99	Metal mass	This work
Cu_5Pt nanoframe	0.5 M H_2SO_4+1.0 M HCOOH	193.85	Pt mass	Ref 13
PtAg nanowire	0.5 M H_2SO_4+1.0 M HCOOH	~185	Pt mass	Ref 56
PtPdCu nanowire	0.5 M H_2SO_4+0.25 M HCOOH	436.2	Pt mass	Ref 29
Pd_3Pt	0.5 M H_2SO_4+0.5 M HCOOH	318	Metal mass	Ref 54
PtAg@Pt	0.5 M H_2SO_4+0.5 M HCOOH	282.6	Pt mass	Ref 19
$Pt_{84}Pb_{16}$ nanoflower	0.5 M H_2SO_4+0.5 M HCOOH	~280	Pt mass	Ref 53
PdFe	0.1 M $HClO_4$+0.1 M HCOOH	352.64	Pd mass	Ref 58
Pt_3Ag	1.0 M $HClO_4$+1.0 M HCOOH	217.56	Pt mass	Ref 14
PtAgCu@PtCu	0.5 M H_2SO_4+0.5 M HCOOH	306	Pt mass	Ref 55
